# Radix *Actinidiae chinensis* induces the autophagy and apoptosis in renal cell carcinoma cells

**DOI:** 10.1186/s40001-024-01881-w

**Published:** 2024-05-19

**Authors:** Biao Liu, Yuanliang Yan, Liang Zhang

**Affiliations:** 1 College of Pharmaceutical Science, Zhejiang University of Technology, No. 18 Chaowang Rd, Gongshu District, Hangzhou, 310014 Zhejiang China; 2 National Clinical Research Center for Geriatric Disorders, Xiangya Hospital, Central South University, Changsha, China; 3 Department of Pharmacy, Xiangya Hospital, Central South University, Changsha, China

**Keywords:** Renal cell carcinoma, Radix* Actinidiaechinensis*, Autophagy, PI3K/AKT/mTOR

## Abstract

**Background:**

Renal cell carcinoma (RCC) is a malignant tumor. Radix* Actinidiae* *chinensis* (RAC) is the root of *Actinidia arguta* (Sieb. et Zucc) Planch. ex Miq. In clinical research, RAC was confirmed to have a certain anti-tumor effect, including liver cancer and cholangiocarcinoma. This study investigated the anticancer effect and mechanism of RAC in RCC cells.

**Methods:**

The 786-O and A498 cells were intervened with varying concentrations of RAC (0–100 mg/mL) to detect the half maximal inhibitory concentration (IC_50_) of RAC. The cells were then co-cultured with 0–50 mg/mL RAC for 0–72 h to assess the effect of RAC on cell viability using the cell counting kit-8. The effects on cell proliferation, cell cycle or apoptosis, migration or invasion, and autophagy were detected using cloning, flow cytometry, Transwell, AOPI assay and Western blot. The number of autophagolysosomes was quantified using a transmission electron microscope. PI3K/AKT/mTOR pathway-related proteins were detected by Western blot. Additionally, an autophagy inhibitor 3-MA was used to explore the underlying mechanism of RAC.

**Results:**

IC_50_ values of RAC in 786-O and A498 were 14.76 mg/mL and 13.09 mg/mL, respectively. RAC demonstrated the ability to reduce the cell malignant phenotype of RCC cells, blocked the S phase of cells, promoted apoptosis and autophagy in cells. Furthermore, RAC was observed to increase autophagy-related proteins LC3II/I and Beclin-1, while decreasing the level of P62. The expression of apoptosis-related proteins was increased, while the ratios of p-PI3K/PI3K, p-AKT/AKT, p-mTOR/mTOR, p-P38/P38 and p-ERK/ERK were reduced by RAC. However, the addition of 3-MA reduced the apoptosis and autophagy- promotion effects of RAC on RCC cells.

**Conclusion:**

RAC induced the apoptosis and autophagy, to inhibit the progression of RCC cells. This study may provide a theoretical and experimental basis for clinical anti-cancer application of RAC for RCC.

## Background

Renal cell carcinoma (RCC) is a highly morbid and metastatic kidney tumor [1–3]. The recognized causes of RCC are hypertension, smoking, and obesity [4]. Studies have found that the effects of radiation therapy, chemotherapy and endocrine therapy in patients with RCC are not satisfactory, and surgery is the only possible cure, but there are still 20% of patients with recurrence [5]. Consequently, the identification of an anti-renal cancer drug with a definitive curative effect will become an important research direction to improve the cure rate of RCC.

The *Radix Actinidiae chinensis* (RAC, also called as Teng ligen in Chinese pinyin name), is the root of *Actinidia arguta* (Sieb. et Zucc) Planch. ex Miq., which contains various bioactivity components, including ursolic acid, quercetin, asiatic acid, and β-sitosterol [6]. In the theoretical system of traditional Chinese medicine (TCM), RAC is sweet, slightly astringent, and has the functions of clearing away heat and detoxifying, diuretic and hemostasis [7]. In clinical research on TCM, RAC was first confirmed to have a certain anti-tumor effect, such as liver cancer and cholangiocarcinoma [8]. Moreover, studies have shown that RAC can achieve the purpose of anti-tumor by inhibiting cell proliferation and invasion, and inducing cell apoptosis [9]. Fang et al. have found that RAC extract attenuates proliferation and metastasis of hepatocellular carcinoma by inhibiting the DLX2/TARBP2/JNK/AKT pathway [10]. Additionally, the ursolic acid, a bioactive ingredient isolated from RAC, has been reported for its autophagy-activating effect in treating osteosarcoma [11].

Autophagy is the self-digestion of cells in autophagolysosomes through various pathways to decompose damaged organelles, proteins and other substances in cells [12]. It has been reported that many anticancer agents induce autophagy and lead to death, which may be an important mechanism by which drugs kill tumor cells [13]. The unbalanced of the PI3K/AKT/mTOR pathway is associated with the development of various malignant tumors [14, 15]. Consequently, molecular targeted therapy focusing on the PI3K/AKT/mTOR pathway has emerged as a hot research topic. Studies have found that the activation of the PI3K/AKT/mTOR pathway plays an important role in the proliferation and migration of RCC cells [16, 17]. Moreover, Punpai et al. have observed that in cancer cells, apoptosis and autophagy were induced by inhibiting the PI3K/AKT/mTOR pathway [18]. Nevertheless, there are still few reports on the subjects of autophagy and PI3K/AKT/mTOR pathway in RCC.

In this study, we aim to observe the effects of RAC on cell viability, apoptosis, migration, invasion, the cell cycle, and autophagy in RCC cells, as well as to explore the autophagy-promoting effect of RAC via the PI3K/AKT/mTOR pathway. To verify the effects of RAC on RCC cells, an autophagy inhibitor, 3-MA, was employed. It hopes to provide some reference and basis for the further research and clinical application of RAC in RCC.

## Methods

### Preparation of RAC extract

RAC was purchased from Hangzhou Huadong Traditional Chinese Medicine Decoction Pieces Co., Ltd (catalogue number: 190211). 1000 g RAC was weighed, dissolved in 5000 mL of ethanol, refluxed for 10 h, extracted 3 times, and the extracts were combined, then evaporated, dried, gradient elution, and the solvent was removed, and the powder was collected and vacuum-dried for 24 h. 100 mL of distilled water was added to 100 g of dry powder, and then the stock solution was diluted to 3.16 mg/mL, 5.62 mg/mL, 10 mg/mL, 15.84 mg/mL, 31.6 mg/mL, 56.23 mg/mL, 100 mg/mL, followed by sterile filtration with a 0.22 μm microporous membrane, divided into packages, and stored in a 4 °C refrigerator for later use.

### Cell culture

The 786-O cell lines (iCell-h235, Homo sapiens, CVCL number: CVCL-1051) and A498 cell lines (iCell-h235, Homo sapiens, CVCL number: CVCL-1056) were obtained and performed a Short Tandem Repeat (STR) profiling from iCell Bioscience Inc (Shanghai, China). 786-O and A498 cells were cultured in RPMI 1640 medium (FI201-01, TransGen Biotech, China) containing 10% fetal bovine serum (FBS), 100 U/mL penicillin–streptomycin at 37 °C and 5% CO_2_. When the cell growth density reached 80%, the old medium was discarded, digested with 0.25% trypsin, and resuspended by adding fresh medium.

### Cell counting kit-8 (CCK-8) assay

The logarithmic phase cell suspension was inoculated into 96-well plates and cultured for 24 h, then grouped into different concentration RAC groups: 0, 3.16, 5.62, 10, 15.84, 31.6, 56.23, 100 mg/mL RAC groups or 0, 1, 5, 10, 15, 30, 50 mg/mL RAC groups. Cells were also divided into different culture time groups: 0, 4, 8, 12, 24, 48, 72 h groups. After dosing with different concentration of RAC or culturing for a corresponding time, 10 μL CCK-8 solution (HY-K0301, MCE, USA) was added and incubated for 2 h. The absorbance at 450 nm was measured, and the cell viability was calculated. Five replicate wells were assayed in parallel.

### Colony assay

The cells in the logarithmic phase were digested, seeded at 500–1000 cells/well in a plate containing 30% FBS in complete medium, and cultured at 37 °C, 5% CO_2_. The medium was changed every 3 days and the cell status was observed. After 2 weeks culturing, photographs were taken when individual cloned cells were large enough to be observed. Then it was stained with 0.1% crystal violet.

### Migration and invasion assay

Matrigel was diluted with a serum-free DMEM high-glycemic culture medium, uniformly coated in the Transwell chamber, and incubated overnight for matrigel flooring. 100 μL cell suspension containing 5 × 10^5^ cells was added to the Transwell chamber for 6 h adherence. After 24 h incubation according to the grouping, matrigel and bottom cells of the upper chamber were washed off, fixed with paraformaldehyde, washed with PBS, stained with crystal violet, then photographed and counted the number of cells migrated and invaded. It was repeated three times.

### Flow cytometry (FCM) assay

The cells in the logarithmic phase were seeded in 6-well plates and grouped into Control group and RAC groups (1, 5, 10 mg/mL RAC) or Control group, 10 mg/mL RAC group, 3-MA group and 10 mg/mL RAC + 3-MA group. 3-MA (S24823) was obtained from Shanghai Yuanye Bio-Technology Co., Ltd (China). After 24 h of treatment, the cells were collected and the cell concentration was adjusted to 1 × 10^6^ cells/mL. 500 μL binding buffer was added and centrifuged to discard the supernatant, and then 100 μL binding buffer was added and mixed. 5 μL Annexin V-FITC and 10 μL PI (556547, BD Pharmingen, USA) were added respectively and reacted at room temperature for 15 min away from light. Finally, 400 μL binding buffer was added, and the apoptosis rate was detected by a flow cytometer (C6, BD, USA).

### Cell cycle assay

The cell suspensions in the logarithmic phase were inoculated into plates, and the culture plates were pre-cultured for 24 h. Then the experiment was carried out according to different doses of RAC (0, 1, 5 and 10 mg/mL RAC). After 24 h of administration, the cell cycle of the cells was detected by flow cytometry.

### Transmission electron microscope assay

The cells were fixed in glutaraldehyde solution and washed 4 times with 0.1 M pH7.0 PBS. It was then fixed with osmic acid and rinsed again. Cells were dehydrated with gradient concentrations of ethanol solution, then treated with 100% ethanol, and then treated with pure acetone. Subsequent gradient permeabilization was performed by using an embedding medium, and then the cells were placed in a 0.5 mL dry tube (pre-filled with about 300 μL of embedding medium), and the polymerizer was heated at 70 °C overnight. The sections were sliced into 70 nm slices by using an ultrathin microtome (EMUC7, Leica, Germany), stained with 100 μL of uranyl acetate 50% ethanol saturated solution for 20 min, rinsed with 100 μL of lead citrate in double distilled water and stained for 15 min, and finally photographed.

### AOPI assay

The AOPI staining was used to detect the effect of the combination of RAC and 3-MA on autophagy in RCC cells. The RCC in the logarithmic phase were placed on the coverslip, and the cells were divided into the Control group, 10 mg/mL RAC group, 3- MA group and 10 mg/mL RAC + 3-MA group and cultured in a cell incubator of 37 °C, 5% CO_2_ for 24 h. It was rinsed 3 times through PBS, dripped with freshly prepared 1 mg/L AOPI staining solution (CA1143, Solarbio, China), incubated at 37 °C, aspirated the AOPI staining solution, and rinsed 3 times with PBS. The slides were mounted with 50% glycerol and placed under an inverted fluorescence microscope to observe the acidic autophagic vesicles.

### Western blot

Firstly, the total protein in RCC was collected and the BCA kit (pc0020, Solarbio, China) was used to detect the total protein. The 10% SDS-PAGE electrophoresis and transfer membrane were performed. The PVDF membranes were blocked with 5% skimmed milk powder for 1.5 h followed by washing with TBST, then it were put into the primary antibody diluent (5% BSA as the diluent) of LC3A/B, Beclin 1, SQSTM1/P62, E-cadherin, Vimentin, Bcl-2, Bax, Cyclin D1, Cleaved-Caspase 3, Pro-Caspase 3, Phospho-PI3K, PI3K, Phospho-pan-AKT1/2/3, AKT2, Phospho-mTOR, mTOR, Phospho-P38 MAPK, P38 MAPK, p-ERK1/2, ERK1/2, β-actin and GAPDH and incubated in 4 °C for 12 h. Then the membranes were washed with TBST and the secondary antibody IgG (H + L) HRP was incubated for 2 h. The ECL was used to detect protein bands, and the protein gray value was calculated by Image J. The details of the antibodies used are list in Table 1.

**Table 1:** Antibodies used for Western blot

Antibody	Species	Dilution	Supplier	Catalogue number
LC3A/B antibody	Rabbit	1:1000	Affinity	AF5402
Beclin 1 antibody	Rabbit	1:1000	Affinity	AF5128
SQSTM1/p62 antibody	Rabbit	1:1000	Affinity	AF5384
E-cadherin antibody	Rabbit	1:1000	Affinity	AF0131
Vimentin antibody	Rabbit	1:1000	Affinity	BF8006
Bcl-2 antibody	Rabbit	1:1000	Affinity	AF6139
Bax antibody	Rabbit	1:1000	Affinity	AF0120
Cyclin D1 antibody	Rabbit	1:1000	Affinity	DF6386
Cleaved-Caspase 3 (Asp175), p17 antibody	Rabbit	1:1000	Affinity	AF7022
Pro-Caspase 3 antibody	Rabbit	1:1000	Abcam	Ab32150
Phospho-PI3K p85 alpha (Tyr607) antibody	Rabbit	1:1000	Affinity	AF3241
PI3K p85 alpha antibody	Rabbit	1:1000	Affinity	AF6241
Phospho-pan-AKT1/2/3 (Ser473) antibody	Rabbit	1:1000	Affinity	AF0016
AKT2 antibody	Rabbit	1:1000	Affinity	AF6264
Phospho-mTOR (Ser2448) antibody	Rabbit	1:1000	Affinity	AF3308
mTOR antibody	Rabbit	1:1000	Affinity	AF6308
Phospho-p38 MAPK (Tyr182) antibody	Rabbit	1:1000	Affinity	AF3455
p38 MAPK antibody	Rabbit	1:1000	Affinity	AF6456
p-ERK1/2 antibody	Rabbit	1:1000	Affinity	AF1015
ERK1/2 antibody	Rabbit	1:1000	CST	4695 s
β-Actin antibody	Rabbit	1:15,000	Affinity	AF7018
GAPDH antibody	Rabbit	1:15,000	Affinity	AF7021
IgG (H + L) HRP antibody	Goat	1:3000	Affinity	S0001

### Statistical analysis

SPSS 20.0 was used for data analysis. One-way ANOVA analysis of variance is used to measure data across multiple groups, and the Tukey or Dunnett’s T3 test is used for comparison between groups. While the Kruskal–Wallis H test is used when the data was not normally distributed. All data were expressed as mean ± standard deviation (SD). *P* < 0.05 suggested that the difference was statistically significant.

## Results

### RAC inhibits the cell viability of RCC cells

**Fig. 1:**
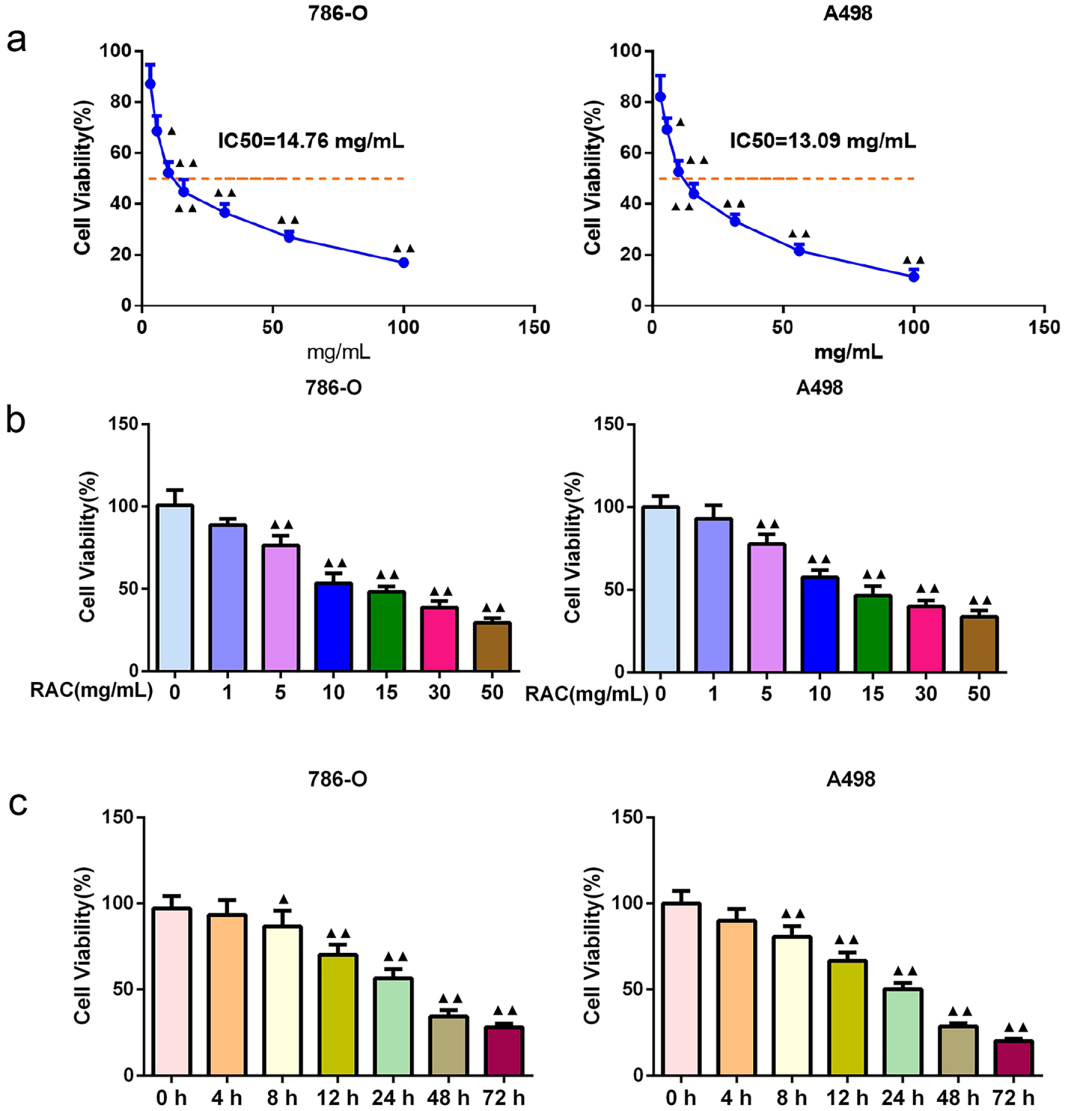
RAC inhibited the 786-O and A498 cell viability. The effects of RAC on RCC cell viability at different concentrations and different incubation times were detected by CCK-8 assay. **a** The half maximal inhibitory concentration (IC_50_) value of RAC on 786-O cells was 14.76 mg/mL, and the IC_50_ value on A498 cells was 13.09 mg/mL. **b **Effects of different concentrations of RAC (0 to 50 mg/mL) on the viability of 786-O and A498 cells. **c** Effects of 10 mg/mL RAC treatment at different times (0 to 72 h) on the viability of 786-O and A498 cells. Data are expressed as mean ± SD, *n* = 5. Compared with the 0 mg/mL group, ^▲^*P* < 0.05, ^▲▲^*P* < 0.01

It can be observed from Fig. 1a, that the cell viability of RCC cells gradually decreased (*P* < 0.01) with the increasing concentration of RAC. The IC_50_ value of RAC on 786-O cells was 14.76 mg/mL, and on A498 cells was 13.09 mg/mL. In addition, from Fig. 1b and c, with the increase in RAC concentration and culture time, the RCC cell viability was decreased gradually.

### RAC inhibited the migration, invasion and cloning ability of RCC cells

**Fig. 2:**
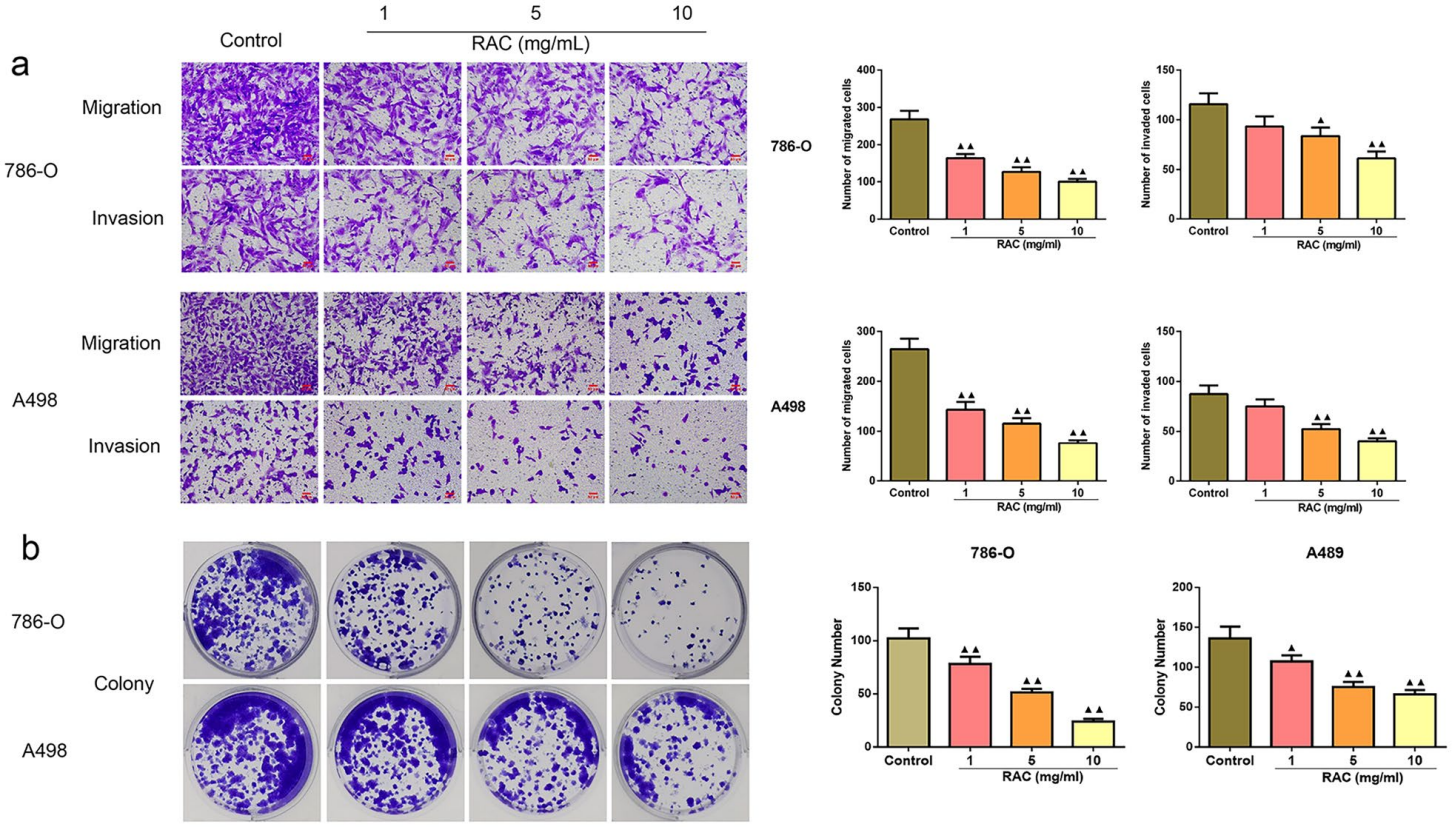
RAC reduced the migration, invasion and cloning abilities of 786-O and A498 cells.The migration, invasion (scale bar = 50 μm) (**a**) and cloning (**b**) abilities of 786-O and A498 cells were detected. Data are expressed as mean ± SD, *n* = 3. Compared with the control group, ^▲^*P* < 0.05, ^▲▲^*P* < 0.01

The results of RAC on cell migration and invasion ability in 786-O and A498 cells were shown in Fig. 2a. Relative to the Control group, the migration numbers of cells in the 1, 5, and 10 mg/mL RAC groups were decreased significantly (*P* < 0.01), the invasion numbers of cells in the 5 and 10 mg/mL RAC groups were decreased significantly (*P* < 0.05). Furthermore, the effect of RAC on cell colonies was shown in Fig. 2b. The results demonstrated that the number of colony cells in the 1, 5, and 10 mg/mL RAC groups was lower than those in the Control group (*P* < 0.05).

### The effect of RAC on RCC cell cycle

**Fig. 3:**
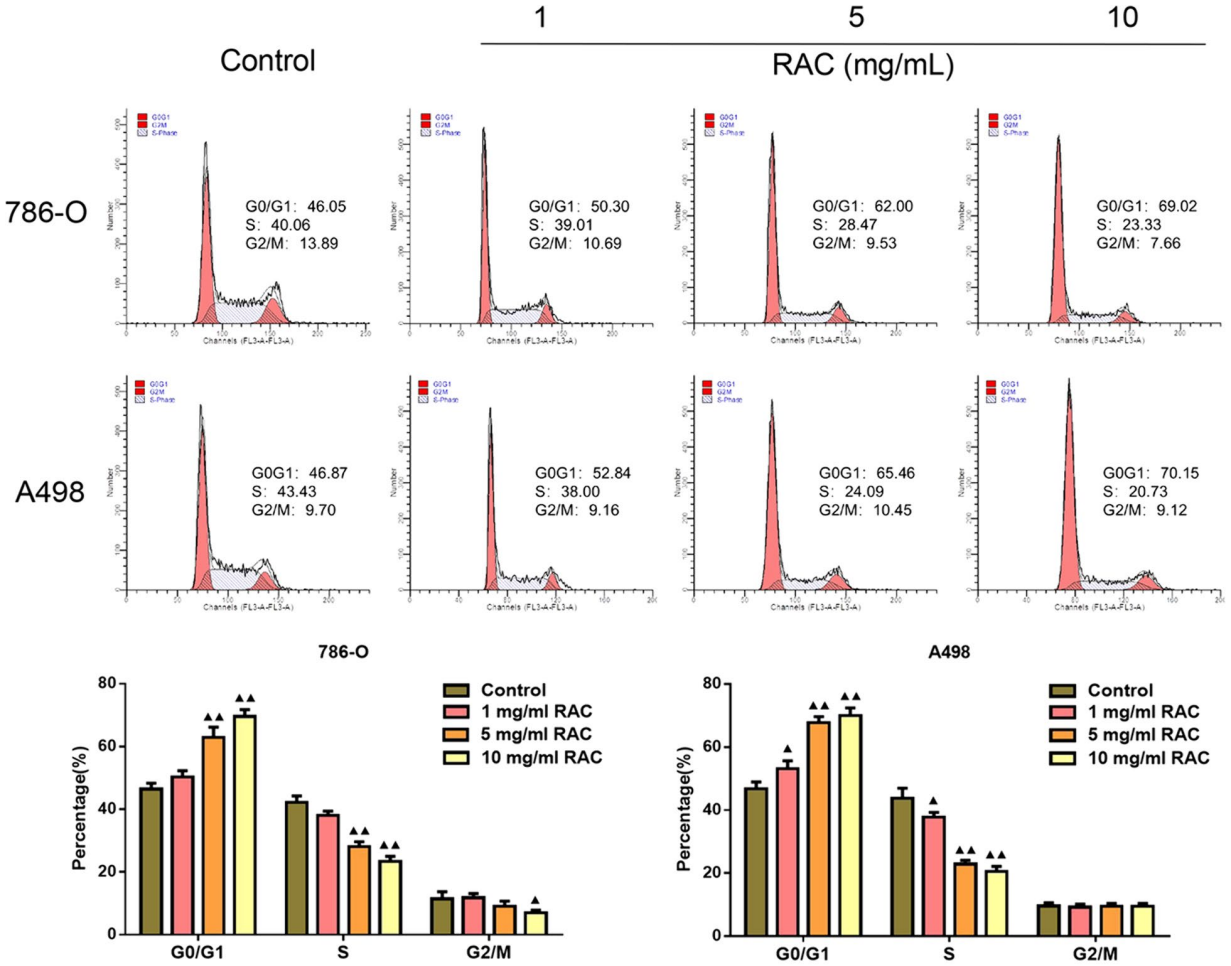
RAC blocked the cell cycle of 786-O and A498 cells. The cell cycle of 786-O and A498 cells was detected by flow cytometry. Data are expressed as mean ± SD, *n* = 3. Compared with the control group, ^▲^*P* < 0.05, ^▲▲^*P* < 0.01

The effect of RAC on the cell cycle of RCC cells was shown in Fig. 3. Compared with the Control group, the 786-O and A498 cells in the RAC medium or high dose groups were increased in the G0/G1 phase, while the number of cells in the S phase was decreased significantly (*P* < 0.05).

### RAC increased the autophagy in RCC cells

**Fig. 4:**
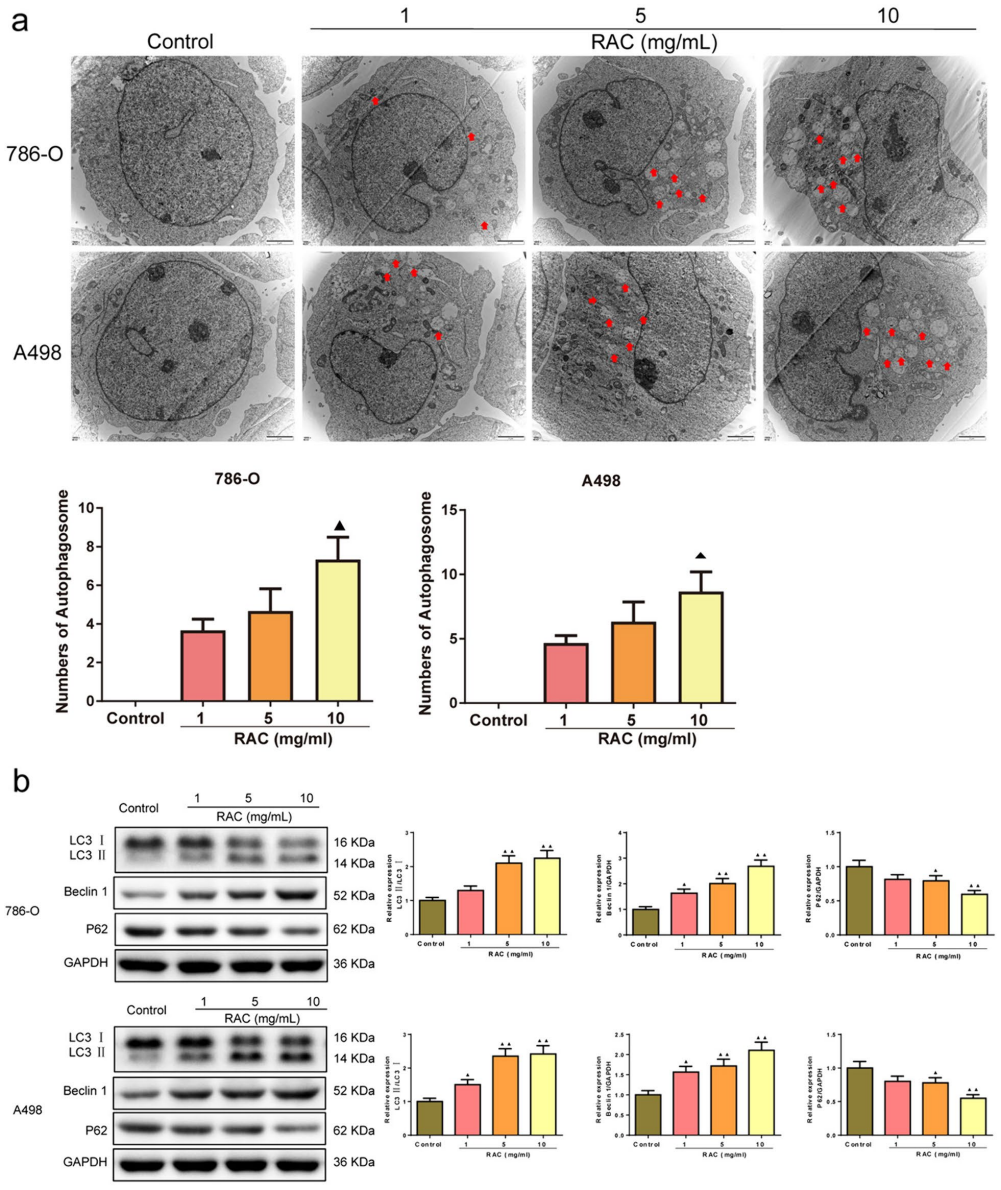
RAC increased the autophagy in 786-O and A498 cells. **a** The number of autophagolysosomes was detected by the transmission electron microscope assay (scale bar = 2 μm). **b** The autophagy-related proteins in 786-O and A498 cells were detected by Western blot. Data are expressed as mean ± SD, *n* = 3. Compared with the control group, ^▲^*P* < 0.05, ^▲▲^*P* < 0.01

Figure 4a showed the effect of RAC on the number of autophagolysosomes in 786-O and A498 cells. Compared to the Control group, following treatment with varying concentrations of RAC, the number of autophagolysosomes in RCC cells was increased, indicating that the autophagy was deepened. Figure 4b illustrated the impact of RAC on autophagy-related proteins. With the increase of RAC concentration, the expression of LC3 and Beclin-1 increased (*P* < 0.05), and the expression of P62 decreased (*P* < 0.05).

### The effect of RAC on the expression of the cell cycle, apoptosis and PI3K/AKT/mTOR pathway-related proteins in RCC cells

**Fig. 5:**
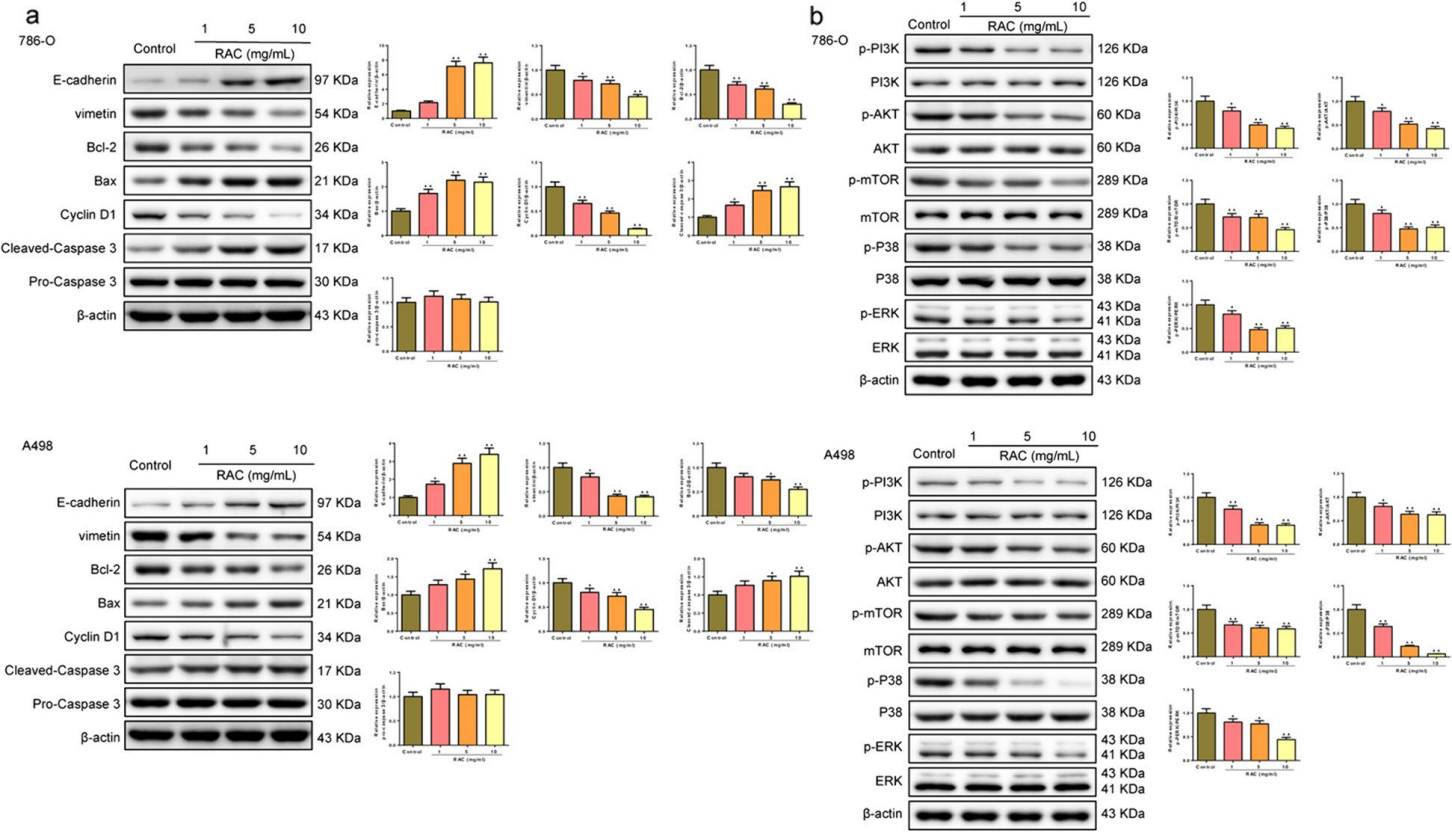
The effect of RAC on the expression of the cell cycle, apoptosis and PI3K/AKT/mTOR pathway-related proteins in RCC cells. The expression of the cell cycle, apoptosis (**a**) and PI3K/AKT/mTOR pathway-related proteins (**b**) in RCC cell were detected by Western blot. Data are expressed as mean ± SD, *n* = 3. Compared with the control group, ^▲^*P* < 0.05, ^▲▲^*P* < 0.01

Figure 5a demonstrated the impact of RAC on the levels of cell cycle and apoptosis-related proteins in 786-O and A498 cells. The levels of E-cadherin, Bax, and Cleaved-caspase3 in RCC cells treated with 1, 5 and 10 mg/mL RAC were higher than those in the Control group, while the levels of vimentin, Bcl-2, and Cyclin D1 were lower. The effect of RAC on the expression of p-PI3K/PI3K, p-AKT/AKT, p-mTOR/mTOR, p-P38/P38, and p-ERK/ERK in RCC cells was shown in Fig. 5b. In comparison to the Control group, the levels of p-PI3K/PI3K, p-AKT/AKT, p-mTOR/mTOR, p-P38/P38, and p-ERK/ERK were found to be lower in RCC cells of 1, 5 and 10 mg/mL RAC (*P* < 0.05).

### 3-MA attenuates the promoting effect of RAC on RCC apoptosis

**Fig. 6:**
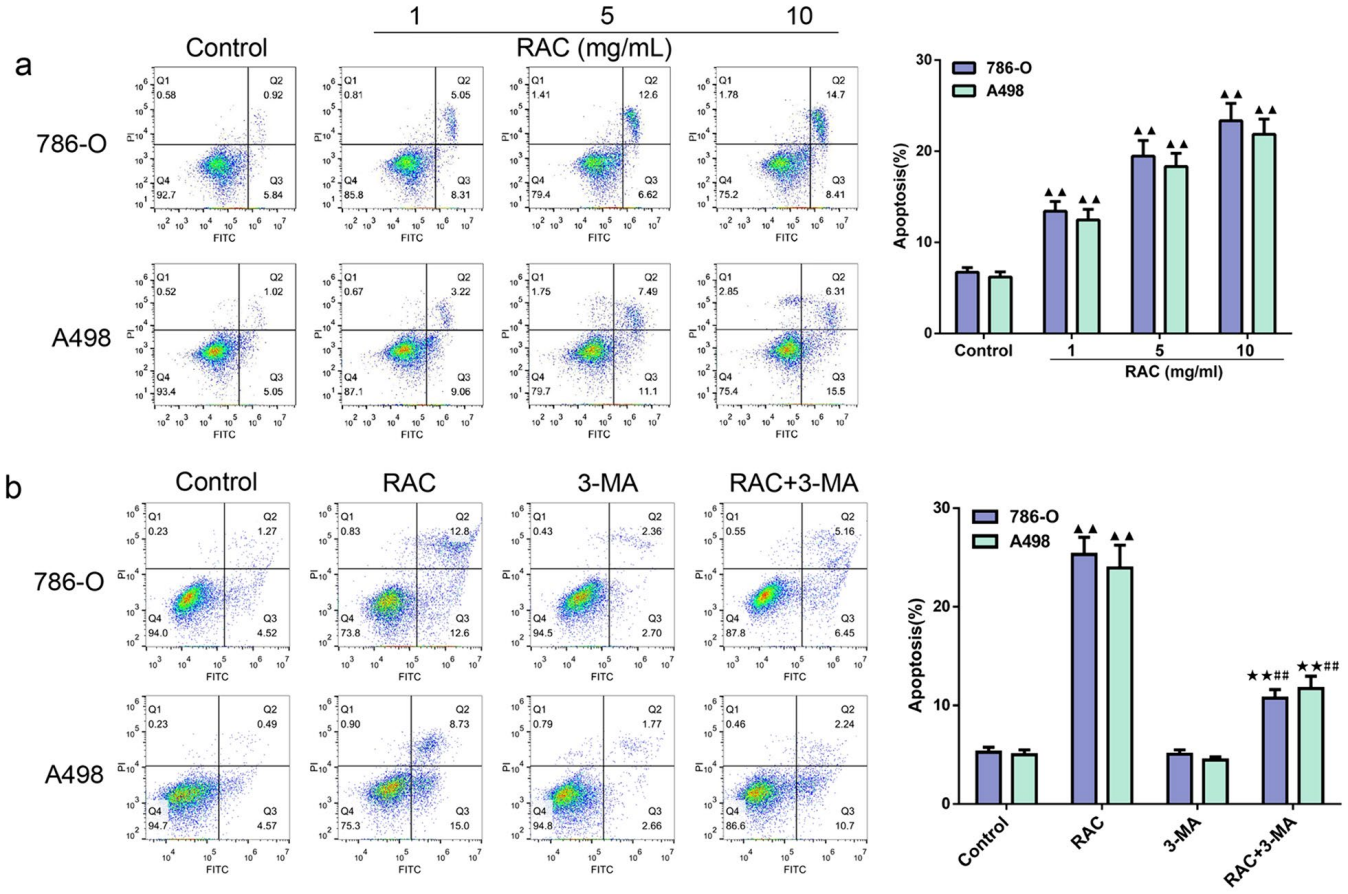
3-MA attenuates the promoting effect of RAC on RCC apoptosis.Effect of RAC and combined with 3-MA on the apoptosis of 786-O and A498 cells was detected by flow cytometry. **a** RAC (1, 5, 10 mg/mL) promoted apoptosis in 786-O and A498 cells. **b** RAC combined with 3-MA attenuated apoptosis of 786-O and A498 cells. Data are expressed as mean ± SD, n = 3. Compared with the control group, ^▲^*P* < 0.05, ^▲▲^*P* < 0.01; compared with the RAC group, ^★^*P* < 0.05, ^★★^*P* < 0.01; compared with the 3-MA group, ^#^*P* < 0.05, ^##^*P* < 0.01

The effect of RAC and 3-MA on the apoptosis of 786-O and A498 cells was shown in Fig. 6a. Compared with the Control group, the apoptosis rates of RCC cells in the low, medium and high dose groups of RAC were found to be higher (*P* < 0.01). The combined effect of RAC and 3-MA on the apoptosis of 786-O and A498 cells was shown in Fig. 6b. The apoptosis rates of RCC cells in the 10 mg/mL RAC were found to be higher (*P* < 0.01) than in those the Control group. Furthermore, with the addition of 3-MA, the apoptosis rates of RCC cells in the RAC + 3-MA group were lower than those in the RAC group, but were higher than the 3-MA group (*P* < 0.01).

### 3-MA attenuates the promotion effect of RAC on RCC cell autophagy

**Fig. 7:**
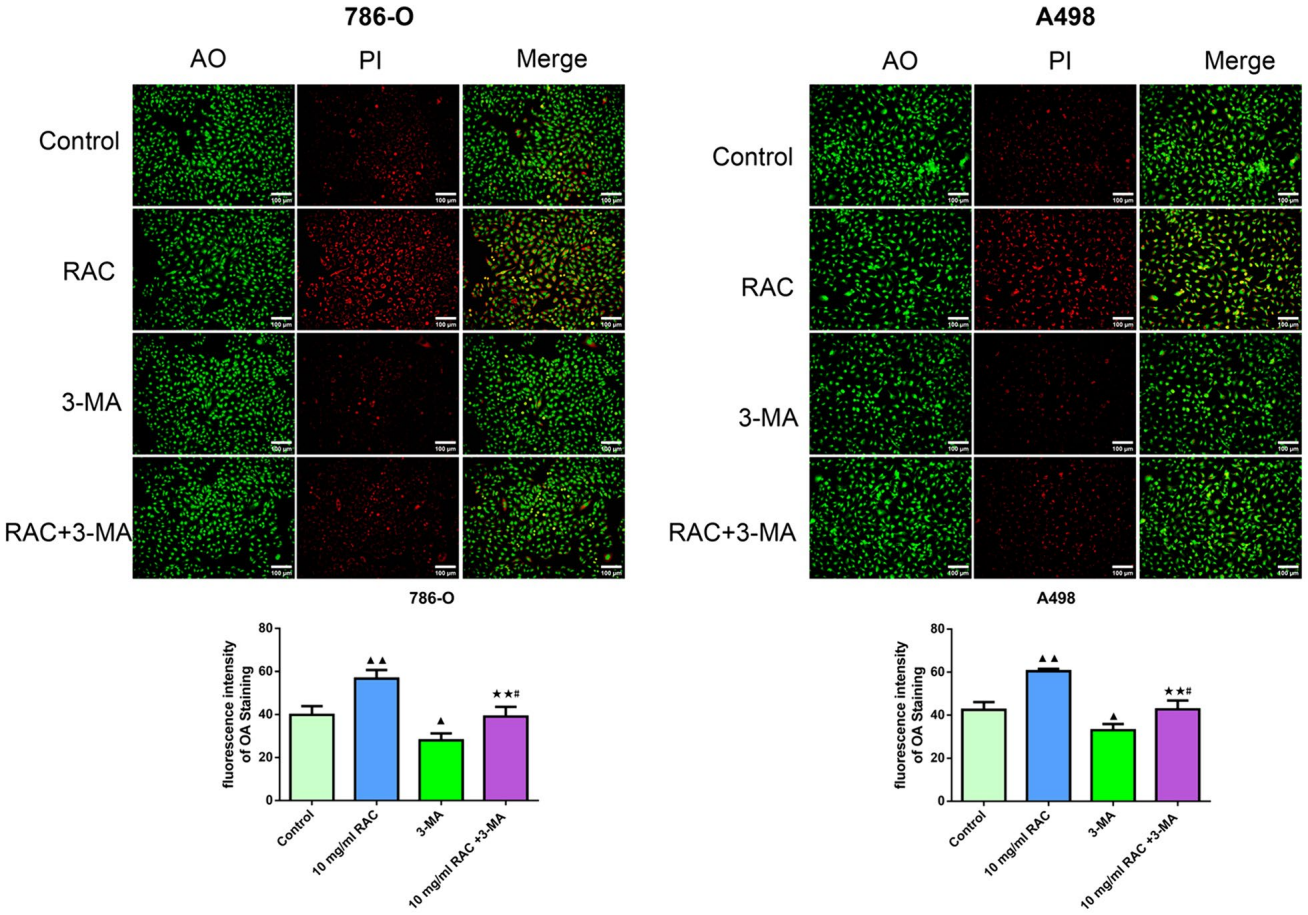
3-MA attenuates the promotion effect of RAC on RCC cell autophagy. AOPI staining was used to detect the effect of RAC and combined with 3-MA on apoptosis of 786-O and A498 (scale bar = 50 μm). Data are expressed as mean ± SD, *n* = 3. Compared with the control group, ^▲^*P* < 0.05, ^▲▲^*P* < 0.01; compared with the RAC group, ^★^*P* < 0.05, ^★★^*P* < 0.01; compared with the 3-MA group, ^#^*P* < 0.05, ^##^*P* < 0.01

Figure 7 illustrates the effect of RAC in combination with 3-MA on the autophagy of 786-O and A498 cells detected using AOPI staining. Compared to the Control group, the RAC group had obvious red fluorescence (*P* < 0.01), while the 3-MA group had less red fluorescence (*P* < 0.05). With the addition of 3-MA, the fluorescence intensity of RCC cells in the RAC + 3-MA group were lower than those in the RAC group, but were higher than that in the 3-MA group (*P* < 0.05).

**Fig. 8:**
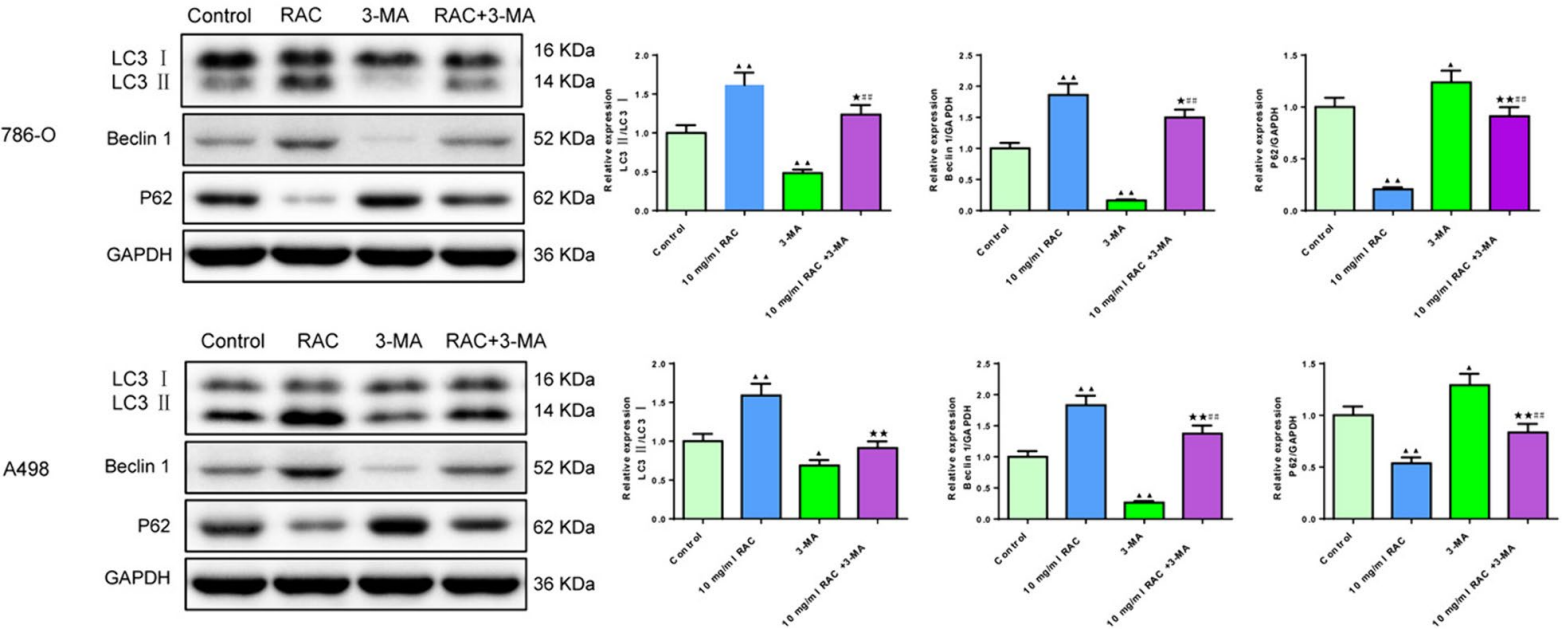
Effects of RAC and combined with 3-MA on the expression levels of autophagy-related proteins in 786-O and A498 cells. The expression of LC3II/I Beclin-1, P62 were detected by Western blot. Data are expressed as mean ± SD, *n* = 3. Compared with the control group, ^▲^*P* < 0.05, ^▲▲^*P* < 0.01; compared with the RAC group, ^★^*P* < 0.05, ^★★^*P* < 0.01; compared with the 3-MA group, ^#^*P* < 0.05, ^##^*P* < 0.01

As shown in Fig. 8, the effect of RAC in 786-O and A498 cells was offset by 3-MA. With the intervention of 3-MA, the levels of LC3 and Beclin-1 in 786-O and A498 cells were decreased (*P* < 0.01 or *P* < 0.05), and the level of P62 was increased (*P* < 0.05) compared to the Control group. Moreover, the levels of LC3 and Beclin-1 in the RAC + 3-MA group were lower than those in the RAC group and the level of P62 was higher than those in the RAC group.

## Discussion

RCC is one of the most common urological malignancies [19]. Recently, the incidence of RCC in China has been increasing, representing a significant threat to human health [20]. In this study, the mechanism of autophagy induced by RAC in 786-O and A498 cells was investigated. The study found that the RAC reduced the activity, cloning ability, migration and invasion ability of 786-O and A498 cells, and the cell cycle was also blocked. Additionally, cell apoptosis and autophagy were activated, accompanied by the regulation of apoptosis and autophagy-related proteins. Moreover, the addition of 3-MA indicated that RAC can induce autophagy and promote apoptosis in RCC cells, further providing a theoretical basis and experimental basis for the anti-renal cancer effect of RAC.

RAC is a Chinese herbal medicine with an inhibitory effect on tumor cells [6]. Studies have found that the RAC can inhibit the proliferation and migration of gastric cancer cells [21]. In this study, we observed that RAC can reduce the viability, proliferation, migration and invasion of RCC cells. Furthermore, Western blot results also showed that RAC altered the expression of proteins involved in apoptosis and the cell cycle, including Bax, cleaved caspase 3, Bcl-2, and cyclin D1. Additionally, it blocked the S-phase of the cell cycle, and promoted cell apoptosis. These findings are consistent with the results of the previous studies, reporting that RAC has a significant inhibitory effect on the proliferation and S-phase number of liver cancer cells, and, as well as the ability to induce cell apoptosis [22, 23].

Autophagy is a process used to remove damaged or redundant macroscopic complexes and organelles in eukaryotic cells, and is involved in the occurrence, development, pathogenesis and metastasis of various malignant tumors [24, 25]. It has been found that the increased protein levels of LC3, Beclin1 and the decreased expression of P62 protein indicated the activation of autophagy [26, 27]. In this study, Western blot results showed that RAC can increase the level of LC3II/I and Beclin1, while decreasing the level of P62. Additionally, the number of autophagic lysosomes was significantly increased, which further enhanced the ability to induce autophagy. This is similar to the anti-cancer mechanism of RAC in breast cancer. It has been reported that RAC can promote the expression of autophagy-related proteins LC3II/I and Beclin1, suppress P62, enhance the autophagy ability and inhibit the proliferation of breast cancer cells [28].

Moreover, in this study, to investigate the effect of RAC on autophagy in RCC cells, the autophagy inhibitor 3-MA was used to inhibit autophagy in RCC cells. It was found that the 3-MA addition could negate the impact of RCA-induced autophagy and apoptosis on RCC cells, indicating that autophagy regulation may be a mechanism through which RAC exerts its anti-RCC effects. Additionally, the RAC + 3-MA group still showed promotion effect of autophagy than the 3-MA alone group, indicating the potential involvement of additional pathways in RAC-induced autophagy. Catechin derivatives, coumarin derivatives and phenolic acid derivatives were identified as the main water-soluble components in RAC [29]. A study found that a coumarin derivative with phenylsulfonylfuroxan, Compound 8b, induces caspase-dependent apoptosis and autophagy in lung adenocarcinoma cells, catechin flavonoid has also been reported to have autophagy induction effect in human glioma cells [30, 31]. These reports also elucidate the pro-autophagic properties of RAC.

The PI3K/AKT/mTOR pathway represents the most significant mechanism of autophagy in cells, and regulating this signaling pathway can affect the occurrence and development of tumors [32]. Wang et al. demonstrated that blocking the PI3K/AKT/mTOR pathway impended the progression of cervical cancer [33]. In addition, Liu et al. found that resveratrol induces autophagy in 786-O cells by inactivating the PI3K/AKT/mTOR signaling pathway [34]. In this study, Western blot results showed that RAC reduced the levels of p-PI3K/PI3K, p-AKT/AKT, p-mTOR/mTOR, p-P38/P38 and p-ERK/ERK in RCC cells. This suggests that the inhibition of the PI3K/AKT/mTOR pathway by RAC induced autophagy in RCC cells. Ye and co-workers have reported that RAC induces autophagy in breast cancer cells by inhibiting the PI3K/AKT/mTOR pathway [35]. These findings align with the results of present study.

Our study investigated the autophagy-promoting effects of RAC on 786-O and A498 cells by inhibiting the PI3K/AKT/mTOR pathway, for the development of a drug to treat renal cancer. However, there are still some limitations to this study. The effects of RAC have not yet been performed in an in vivo RCC animal model. Further, a deeper molecular mechanism exploration will be carried out in the future research.

## Conclusions

In conclusion, the anti-RCC effect of RAC was investigated in this study through investigation of its effects on 786-O and A498 cells. It was demonstrated that RAC reduced the cell activity, colony ability, migration and invasion ability of 786-O and A498 cells, and the cell cycle was also blocked, cell apoptosis and autophagy were activated. Further study suggested that this anti-RCC effect may be related to the autophagy regulation and the PI3K/AKT/mTOR pathway. Our study expounded the anti-renal cancer activity and mechanism of RAC at the molecular level; however, it is not sufficient for the clinical treatment and further research will be conducted in animals to provide a theoretical and experimental basis for further study and clinical treatment of RCC.

## Data Availability

The data supporting the conclusions of this article is included within the article.
